# Epidemiological and clinical characteristics of esophageal carcinoma: An experience from tertiary care hospital of Karachi, Pakistan

**DOI:** 10.12669/pjms.40.1.7419

**Published:** 2024

**Authors:** Nazish Butt, Sabir Ali Soomro, Ghulam Haider, Aemon Zehra

**Affiliations:** 1 Nazish Butt, MBBS, FCPS. Associate Professor and Head of Gastroenterology, Department of Gastroenterology, Jinnah Postgraduate Medical Center (JPMC), Karachi, Pakistan; 2 Sabir Ali Soomro, MBBS. Postgraduate trainee (FCPS Gastroenterology & Hepatology), Department of Gastroenterology & Hepatology, Jinnah Postgraduate Medical Center (JPMC), Karachi, Pakistan; 3 Ghulam Haider, MBBS, FCPS. Associate Professor, Department of Oncology, Jinnah Postgraduate Medical Center (JPMC), Karachi, Pakistan; 4 Aemon Zehra, MBBS. Postgraduate trainee (FCPS Gastroenterology & Hepatology), Department of Gastroenterology & Hepatology, Jinnah Postgraduate Medical Center (JPMC), Karachi, Pakistan

**Keywords:** Squamous cell carcinoma, Adenocarcinoma, Betel nuts Pakistan

## Abstract

**Objectives:**

To describe current trends and characteristics of esophageal cancer (EC) over the past ten years largest tertiary care hospital of Karachi, Pakistan.

**Methods:**

This single center retrospective study was conducted at the Department of Gastroenterology and Oncology, Jinnah Postgraduate Medical Center, Karachi, Pakistan, between the period of ten years from 1^st^ January 2010 to 31^st^ December 2019. Patient data including epidemiological characteristics such as age, gender, education, residence, occupation, addictions, comorbidities, symptoms, location of EC, laboratory parameters and histopathological type were recorded. All patients with missing histological and radiological findings were excluded.

**Results:**

The mean age of all patients was 49.26±14.24 years and among them majority were females 566 (56.1%). Almost, 834 (82.7%) patients had SQC and 175 (17.3%) presented with ADS. Most common presenting symptom was dysphagia in both groups 327 (32.4%) followed by vomiting (22.8%) and weight loss 196 (19.4%). The Esophagogastroduodenoscopy (EGD) findings showed the distal esophagus involvement in most of the patients (36.3%) in both groups. CT scan findings showed that the lower region was the most involved region in 367 (36.4%) patients, followed by middle and upper in 227 (22.5%) and 156 (15.5%) patients respectively. The thickening of mass on CT- scan was circumferential in most of the patients (42.7%) in both groups.

**Conclusion:**

According to our findings, SQC is more prevalent than ADC. Female predominance especially at younger age was most common as compared to past studies. No significant association between a single risk factor has been found in our study however smoking and betel nut chewing were found as known putative risk factors.

## INTRODUCTION

Esophageal carcinoma (EC) ranks the eighth most prevalent cancer worldwide.^1^ EC is solely responsible for 7% of gastrointestinal cancers. EC is categorized into two variants, adenocarcinoma (AD) and squamous cell carcinoma (SQC), later one is 90% prevalent and has higher incidence in regions of Asia.^2^ Age-standardized rate (ASR) opted for cases of EC worldwide reports 7.7 per 100,000 cases for Asia with maximum ASR of 12.5-12.7 per 100,000 cases in Bangladesh and China while ASR reported for Pakistan is 4.1 per 100,000 cases.^3^ EC has increased gender predilection towards male gender affecting 2-4 times more frequently as compared to females worldwide.^4^,^5^

Multiple risk factors are associated with EC including Barrett’s esophagus, obesity, and chronic Gastro-esophageal reflux disease (GERD), alcohol prevalent in the western population while smoking, opium, hot beverages, inappropriate use of salt, nutritionally deficient diet, low socioeconomic status, betel nut chewing, human papilloma virus and family history of cancer are factors prevalent in Asian EC belt.^6^ Dysphagia is the most common symptom of EC followed by weight loss, odynophagia, hoarseness of voice, retrosternal burning pain, anemia and blood in vomitus.^7^ Given this and the fact that the incidence of esophageal cancer is on the rise, further details of this malignancy are required, especially squamous cell carcinoma. To our knowledge, there is limited literature available on the characteristics of EC, specifically for Pakistani population.

Thus, we aim to conduct a study to describe characteristics of EC over the past ten years, in a largest tertiary care hospital of metropolitan city of Pakistan to better understand the various modes of presentation of this disease for early diagnosis as it has dismal prognosis.

## METHODS

It was a single center retrospective study conducted in the department of Gastroenterology and Oncology, Jinnah Postgraduate Medical Center, Karachi, Pakistan.

### Ethical Approval

It was obtained from institutional ethical review board (NO.F.2-81/2022-GENL/282/JPMC).

### Inclusion & Exclusion Criteria

All patients with EC presented from 1^st^ January 2010 to 31^st^ December 2019 were included. All data was extracted from the data base of the hospital with the permission of respective department. Patients who had incomplete records were excluded from the study. Patient data including epidemiological characteristics such as age, gender, education, residence, occupation, addictions, comorbidities, symptoms, location of EC, laboratory parameters and histopathological type were recorded in proforma. The laboratory investigations such as complete blood counts were also recorded. Similarly, histopathology, EGD and radiological findings were also collected.

### Statistical analysis

The Statistical Package for Social Science (SPSS version 25.0) was used for data analysis. The normality of the data was measured by Shapiro Wilk test. Independent sample t-test was used to find difference between numeric variables. The chi-square test was analyzed for categorical variables. P-values < 0.05 were deemed significant.

## RESULTS

The mean age of all patients was 49.26±14.24 years and among them majority were females 566 (56.1%) and residents of Sindh province 849 (84.1%). The smoking was the most common addiction followed by betel leaf and betel nuts among patients. 88% of the patients were hypertensive and 5.3% were diabetics. Almost, 834 (82.7%) patients had SQC and 175 (17.3%) presented with ADC. All details regarding demographic profile and types of EC are presented in Table-I. Most common presenting symptom was dysphagia in both groups 327 (32.4%) followed by vomiting (22.8%) and weight loss 196 (19.4%). Retrosternal burning, acid reflux, and odynophagia were significantly more prevalent in patients with SQC as compare to ADC (P-value= <0.05) as mentioned in Table-II. Hemoglobin level was 11.01 ± 1.65 and 11.51 ± 2.02 for SQC and ADC that were significantly low in patients with SQC (P= 0.003). However, patients with ADC had low platelet count as compared to patients with SQC, (P-value 0.03). Table-II.

**Table-I:** Demographic characteristics and Types of EC.

S.No	Variables	Characteristics	N (%)
1	Gender	Male	443 (43.9%)
		Female	566 (56.1%)
2	Residence	Sindh	849 (84.1%)
		Baluchistan	62 (6.1%)
		Punjab	18 (1.8%)
		Khyber Pakhtunkhwa	43 (4.3%)
		Gilgit Baltistan	18 (1.8%)
		Afghanistan migrants	16 (1.6%)
		Others	3 (0.3%)
3	Ethnicity	Sindhi	434 (43.0%)
		Balochi	90 (8.9%)
		Punjabi	55 (5.5%)
		Balti	12 (1.2%)
		Pashtun	108 (10.7%)
		Urdu	259 (25.7%)
		Others	51 (5.1%)
4	Occupation	Housewife	560 (55.5%)
		Labor	208 (20.6%)
		Farmer	74 (7.3%)
		Government employee	18 (1.8%)
		Private business	58 (5.8%)
		Shopkeeper	14 (1.4%)
		Retired	28 (2.8%)
		Others	49 (4.9%)
5	Comorbidities	None	834 (82.7)
		Diabetes Mellitus	53 (5.3%)
		Hypertension	88 (8.7%)
		Ischemic heart disease	31 (3.1%)
		Tuberculosis	5 (0.5%)
		Hepatitis B	9 (0.9%)
		Hepatitis C	26 (2.6%)
		Others	18 (1.8%)
6	Addictions	None	471 (46.7%)
		Smoking	150 (14.9%)
		Hookah	23 (2.3%)
		Betel leaf	128 (12.7%)
		Betel nuts	91 (9.0%)
		Betel quid (Gutka)	90 (8.9%)
		Naswar	82 (8.1%)
		Alcohol	5 (0.5%)
		Other illicit drugs	5 (0.5%)
7	Histopathology	Squamous cell carcinoma	834 (82.7%)
		Adenocarcinoma	175 (17.3%)

**Table-II:** Symptoms amongst the histopathological variants of the study population (N = 1009).

S. No.	Clinical feature	SQC n=834	ADC n=175	p-value
1	Fever 25 (2.5%)	19 (2.3%)	6 (3.4%)	0.419**
2	Weight loss 196 (19.4%)	164 (19.7%)	32 (18.3%)	0.675*
3	Upper GI bleeding 47 (4.7%)	41 (4.9%)	6 (3.4%)	0.396*
4	Retrosternal burning 60 (5.9%)	42 (5.0%)	18 (10.3%)	0.008*
5	Acid reflux 22 (2.2%)	22 (2.6%)	0 (0.0%)	0.022**
6	Dysphagia 327 (32.4%)	260 (31.2%)	67 (38.3%)	0.068*
7	Odynophagia 45 (4.5%)	45 (5.4%)	0 (0.0%)	0.002*
8	Anorexia 4 (0.4%)	2 (0.2%)	2 (1.1%)	0.141**
9	Sticking of food 7 (0.7%)	7 (0.8%)	0 (0.0%)	0.612**
10	Regurgitation of food 15 (1.5%)	11 (1.3%)	4 (2.3%)	0.310**
11	Nocturnal cough 33 (3.3%)	30 (3.6%)	3 (1.7%)	0.203*
12	Vomiting 230 (22.8%)	189 (22.7%)	41 (23.5%)	0.826*
13	Dyspnea 2 (0.2%)	1 (0.1%)	1 (0.6%)	0.317**
14	Neck swelling 1 (0.1%)	1 (0.1%)	0 (0.0%)	1.000**
15	Halitosis 1 (0.1%)	1 (0.1%)	0 (0.0%)	1.000**
16	No symptoms46 (4.6%)	43 (5.2%)	3 (1.7%)	0.047*

* Indicates the chi-square test used to compute the p-value,

** Indicates Fisher’s exact test to compute the p-value.

The EGD findings showed that most of the patients had distal esophageal mass (36.3%) in both groups followed by middle and middle to distal in 36.3% and 16.2% respectively. According to characteristics of mass on EGD was ulcerated in 32.2% of the patients followed by circumferential in 25.6% of them. Other characteristics of mass and their distribution in both groups are shown in Table-III. The luminal narrowing was present in 801 (79.4%) of the patients and higher in SQC (80.3%) than ADC (74.9%). However, there was no statistically significant different found between two groups with P-value of 0.21.

**Table-III:** Correlation of biochemical markers amongst the variants of EC n=1009.

S. No.	Laboratory investigations	Value	SQC (n=834)	ADC (n=175)	p-value
1	Hemoglobin (g/dl)	11.10 ± 1.73	11.01 ± 1.65	11.51 ± 2.02	0.003
2	Mean corpuscular volume (fL)	80.62 ± 9.08	80.31 ± 8.79	82.10 ± 10.28	0.034
3	Platelets (10^9^cells per liter)	253.64 ± 107.95	257.97 ± 107.38	232.97 ± 108.58	0.006
4	Total leucocyte count (10^9^cells per liter)	7.26 ± 3.46	7.32 ± 3.48	6.97 ± 3.36	0.229

Similarly, CT scan findings showed that the lower region was the most involved region in 367 (36.4%) patients, followed by middle and upper in 227 (22.5%) and 156 (15.5%) patients respectively. This distribution was same for both groups (SQC and ADC) as mentioned in Table-III. The ulcerated circumferential and irregular mass showed significant difference between SQC and ADC (P- value= 0.002, 0.009 respectively). The thickening of mass on CT- scan was circumferential in most of the patients (42.7%) in both groups. However, 358 (35.5%) showed circumferential and mural thickening followed by mural in 119 (11.8%) patients. The circumferential and mural with circumferential showed significant difference between SQC and ADC (P- value < 0.001) as mentioned in Table-III.

## DISCUSSION

World-wide, SQC and ADC were most common cancers of esophagus and constituted 95% of all EC cases. Overall, EC has a unique regional distribution, with South and Central Asian countries making up the “Asian cancer belt “have an extremely high rates, with squamous cell being the most prevalent subtype in the area. Prevalence of esophageal cancer is high in Pakistan, accounting for 5% of all cancers in men.^8^

In our study mean age of all patients was 49.26±14.24 years which was contrary to previous studies conducted in neighboring countries, which revealed age 60±10 years as the most common age group for both SQC & ADC of the esophagus 9,10. EC therefore, is a disease of younger age group in our country. A study conducted in Karachi, during 1995-2002, 11 to identify trends of EC showed the ASIRs in men remained consistent during the past ten years, whereas an increasing tendency was seen in the ASIRs in women, this is inconsistent with our study, as majority subjects were females 566 (56.1%), however studies from US reports Male to female ratio (3: 1)^12^

SQC was the most common subtype EC among both genders found in 82.7% of the patients and only 17.3% had EC-ADC, this data was consistent with findings from most of the Asian countries, but ADC still predominates in the west.^13^ Even though SQC was the most prevalent histology in our study, the most common location was the lower esophagus, 367 (36.4%). This indicates that Barrett’s esophagus was not likely to be the cause of esophageal cancer in our community. Endoscopically mass appeared ulcerated in 32.2% of the patients, followed by circumferential involvement in 25.6% but it has no obvious histological or prognostic correlation between the two groups. The most common presenting symptom was dysphagia in both groups 327 (32.4%) followed by vomiting (22.8%) and weight loss 196 (19.4%) similar to data worldwide.^14^

Smoking and alcohol both have been reported as significant risk factors for EC in Western and Asian countries^15^. In our region the incidence of esophageal SQC has remained relatively on rise thus to investigate possible reasons for this diverging incidence rate we analyzed data and found that in our study majority 471(46.7%) had no identifiable cause and smaller fraction of patients smoked cigarettes whereas none of the patients had ever used alcohol which is in accordance with studies conducted among central Asian cancer belt.^16^ The majority of the elements that have been identified as contributing to the disease’s etiology are linked with personal behaviors like alcohol, cigarette use and betel nut chewing that appears to have an esophageal rather than a systemic effect; this may explain the elevated risk of SQC. This may imply that the disease can be prevented by primary means.

### Limitations

Our study was hindered by multiple limitations. The first limitation is the retrospective nature of our study, which carries with its inherent weaknesses in design when compared to prospective studies. Since, current study was conducted in a single institution, therefore it was not possible to examine all the risk factors associated with development of EC. There was lack of metastatic workup, follow up after treatment and prognostic data was missing. Therefore, we suggest further large-scale epidemiological studies to elaborate about all the risk factors and to explore the relationship between tumor stage and risk factors.

## CONCLUSION

The majority of EC patients were young females, with SQC being the most common histologic subtype. Thus high suspicion of underlying EC should be kept in mind even in young patients. Healthcare policies need to be established at the national level for encouraging lifestyle modifications, dietary changes and avoidance of habits such as cigarette smoking, consumption of betel nuts and betel quid, for possible reduction in the disease burden.

### Authors’ contributions:

**NB:** Designed this study, are responsible and accountable for the accuracy and integrity of the work.

**NB** and **SS:** Wrote the initial draft of the study.

**GH** and **AZ:** Involved in the data collection and data analysis. All authors have approved final draft of the study.
